# Surgical treatment for renal masses in the elderly: analysis of oncological, surgical and functional outcomes

**DOI:** 10.1590/S1677-5538.IBJU.2018.0310

**Published:** 2019-07-27

**Authors:** Slawomir Poletajew, Piotr Zapała, Bartlomiej Kopczyński, Lukasz Białek, Sylwia Bender, Tomasz Mutrynowski, Mateusz Nowak, Julia Mróz, Grzegorz Pędzisz, Bartosz Dybowski, Piotr Radziszewski

**Affiliations:** 1 Department of Urology, Medical University of Warsaw, Warsaw, Poland

**Keywords:** Kidney Neoplasms, Survival, Delayed Graft Function

## Abstract

**Purpose:**

Radical treatment in elderly patients with renal tumor remains debatable due to uncertainties regarding the risk of surgical complications, risk of end-stage renal disease (ESRD) and survival benefit. The aim of the study was to assess outcomes of radical treatment for renal cancer in elderly patients.

**Materials and Methods:**

This retrospective analysis enrolled 507 consecutive patients treated with partial or radical nephrectomy due to renal mass. Patients with upfront metastatic disease (n=46) and patients lost to follow-up (n=110) were excluded from the analysis. Surgical, functional (screen for ESRD development) and survival outcomes were analyzed in patients aged >75 years in comparison to younger individuals.

**Results:**

The analyzed group included 55 elderly patients and 296 younger controls. Within the cohort a total of 148 and 203 patients underwent radical and partial nephrectomies respectively. The rate of surgical complications, including grade ≥3 Clavien- Dindo complications, did not differ between groups (3.6% vs. 4.4%, p=0.63). Median length of hospital stay was equal in both groups (7 days). During a follow-up (median 51.9 months, no difference between groups), ESRD occurred in 3.4% of controls and was not reported in elderly group (p=0.37). Younger patients demonstrated a statistically significant advantage in both overall survival and cancer-specific survival over elderly patients (OS 94.6% vs. 87% p=0.036, CSS 97.3% vs. 89.1% p=0.0008).

**Conclusions:**

Surgical treatment in elderly patients with renal tumor is as safe as in younger individuals and does not increase the risk of ESRD. However, cancer specific survival among these patients remains shorter than in younger patients.

## INTRODUCTION

Incidence of renal cell carcinoma (RCC) has been constantly increasing in last decade (1). In 2012, RCC represented the ninth most common malignancy worldwide (2). The rising incidence of renal cell carcinoma is attributed to improved imaging facilitating incidental detection of smaller renal masses. However, its rising prevalence results also from the increase in elderly population.

According to the clinical guidelines of the European Association of Urology (EAU), surgery with curative intent remains standard treatment option for patients with renal cancer. However, radical management can constitute a challenge in elderly patients as impairment of kidney function, surgical complications and long postoperative recovery are believed to be more likely to occur in these patients. At the same time, since progression of renal tumors is in general slow, it is unclear whether elderly patients live long enough to benefit from surgery in terms of overall- and cancer-specific survival. Foregoing controversies comprise rationale for active surveillance as alternative for partial and radical nephrectomy in elderly patients with small renal masses.

The aim of this retrospective study was to assess perioperative, functional and oncologic outcomes of radical treatment of renal tumors in elderly patients with a comparison to younger individuals.

## MATERIAL AND METHODS

### Patients

**Figure 1:**
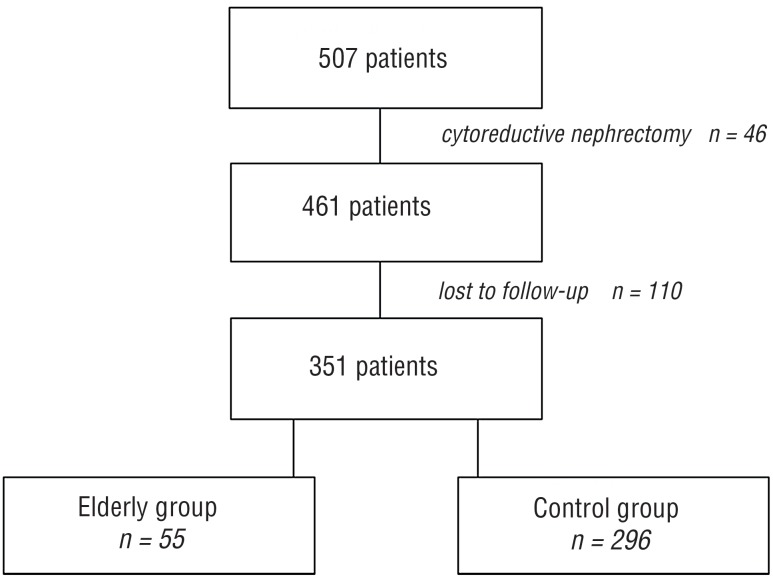
Patients analyzed in the study - flowchart diagram.

Five hundred and seven consecutive patients with renal tumor who underwent surgical treatment from 2010 to 2015 in a single department were enrolled into this retrospective analysis. Patients with upfront metastatic disease (n=46) and patients lost in follow-up (n=110) were excluded from the final analysis. Remaining 351 patients were stratified depending on age into two groups: aged 75 or older (elderly group, n=55) and aged <75 years (control group, n=296). Both radical nephrectomies (RN, n=148) and partial nephrectomies (PN, n=203) were analyzed. Flow diagram presenting study population is presented in the Figure-1.

### Methods

Patient data were gathered for specified demographic and clinical variables, tumor characteristics, perioperative, and oncologic outcomes. Pathologic data comprised histological type and subtype (3), stage (assigned according to the 2009 TNM classification system) (4), grade (5). Perioperative outcomes included ischemia time for PN, length of hospital stay (LOS), transfusion rate, and surgical complications graded according to the Clavien - Dindo classification (6). Postoperative follow-up included ultrasound or CT imaging, performed every six months assessed with telephone survey. Study end-points were overall survival, cancer-specific survival, surgical complications and renal functional outcomes defined by development of established diagnosis of end-stage renal disease (ESRD) (chronic renal disease requiring dialysis).

### Statistical analysis

Continuous variables were presented as medians accompanied by ranges or interquartile ranges (IQR). Differences between groups were evaluated using the U Mann-Whitney test for continuous variables, and by the chi-square test for categorical variables. Overall survival (OS) and cancer-specific survival (CSS) were estimated using the Kaplan-Meier method. Before survival analysis, clinical and pathological comparison between age groups were performed to detect potential confounders. Differences in survival were assessed using the log-rank test. To support rationale for subgroup analysis Cox proportionalhazards analysis was implemented. For all statistical analyses, a 2-sided P value <0.05 was considered statistically significant. Statistical analyses were performed using SAS 9.4.

## RESULTS

### Clinical and pathologic data

Median age in the cohort was 63 years (IQR=17). In 299 patients (88.2%) renal cell carcinoma was found. Prevalence of clear-cell (ccRCC, 71.7% vs. 69.6%), chromophobe (chRCC, 1.9% vs. 4.2%) and papillary RCC (pRCC, 9.0% vs. 15.4%) did not differ between the elderly and control group. Clinical and pathologic data in the study groups are summarized in Table-1. In patients who underwent radical nephrectomy symptomatic renal tumor and ≥T2 lesions were significantly more common (p<0.0001). In RN group tumors were also larger (p<0.0001) and preoperative creatinine was higher (p=0.003).

**Table 1:** Clinical and pathologic data in elderly and control group.

Variable		Elderly group (n= 55)	Control group (n= 296)	P
**Male**				
(no. / %)		31 / 56.4%	183 / 61.8%	NS
**Age**				
(years, median / IQR)		80 / 5	50 / 11	**<.0001**
**Symptomatic** *				
(no. / %)		17 / 23.5%	63 / 34.7%	NS
**Largest lesion diameter**				
(cm, median/ IQR)		4 / 3.5.	3 / 2	NS
**Endophytic**				
(no. / %)		7 / 20.6%	34 / 18.5%	NS
**Clinical staging**				
(no. / %)	T1	37 / 84.1%	219 / 86.2%	NS
	T2-3	7 / 15.9%	35 / 13.8%	
**Histopathologic type**				
(no. / %)	RCC	44 / 83.0%	255 / 89.2%	NS
	Other	9 / 17.0%	31 / 10.8%	
**BMI**				
(kg/m^2^, median/IQR)		27.2 / 9.4	27.5 / 21.8	NS
**Preoperative creatinine**				
*(mg%, median/IQR)*		1.1 / 0.4	1.0 / 0.3	**0.005**
**Partial nephrectomy**				
(no. / %)		22 / 56.4%	181 / 73.3%	**0.037**
**Follow-up**				
(months, median/ IQR)		53 / 32.3	51.6/ 34.3	NS

**NS** = not significant; **IQR** = interquartile range; **BMI** = body mass index;

* -presence of any symptoms suggestive for renal tumor including hematuria, palpable tumor, flank pain

### Survival analysis

**Figure 2:**
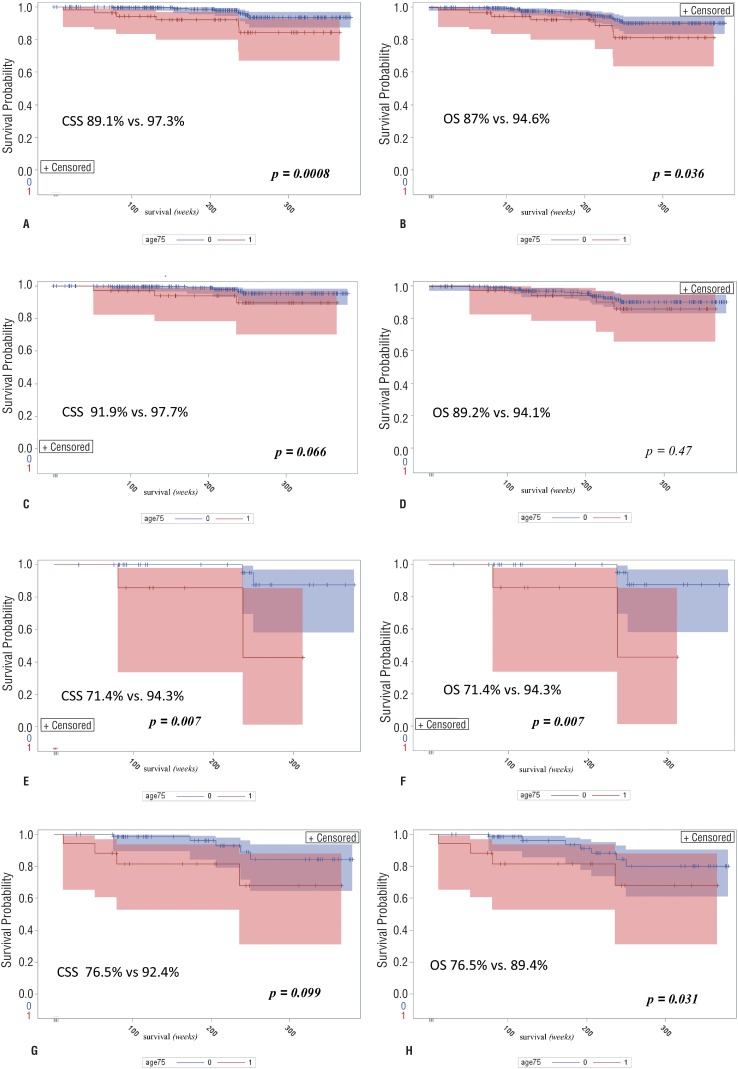
Cancer-specific survival (CSS) and overall survival (OS) analysis - Kaplan-Meier curves.

Median follow-up was 51.9 months (IQR=34.3). In analyzed period a total of 24 deaths, including 14 (58.3%) deaths caused by RCC progression, were reported. Six out of 55 (10.9%) and eight out of 296 (2.7%) patients died due to cancer in elderly and control group, respectively. Younger patients demonstrated a statistically significant advantage in overall survival and cancer-specific survival over elderly patients (CSS 97.3% vs 89.1%; OS 94.6% vs 87%; Figure 2A-B). Multivariable Cox proportional-hazards analysis (Table-2) confirmed type of surgery (partial vs radical nephrectomy) and age group as factors associated with cancer-specific survival. When survival analysis was restricted to T1a and T1b tumors, cancer-specific survival in elderly group and control group was 91.9% and 97.7%, respectively. Although some tendency can be noted, survival advantage of the control group failed to achieve statistical significance (Figure-2C). Similarly, no statistical significance was achieved regarding overall survival (OS 89.2% vs 94.1%; Figure-2D). In T2-T3 tumors, CSS was significantly inferior in elderly group (71.4% vs 94.3%; Figure-2E). All deaths reported in T2-T3 were cancer-related (OS was equal to CSS).

**Table 2:** Mutivariative analysis - Cox proportional - hazards model.

Factor	Hazard ratio	P
Partial nephrectomy	3.404	0.0440
Age 75 or older	0.055	0.0057

No death was noted in elderly patients treated with partial nephrectomy. When analyzed exclusively for PN, CSS and OS in control group were 99.5% and 96.1% respectively. Survival advantage in the elderly group was not significant. On the contrary, in patients treated with RN, CSS was higher in control group then in elderly group (92.4% vs 76.5%; Figure-2G) as well as OS (89.4% vs 76.5%; Figure-2H).

### Surgical complications and renal function outcomes

Four (7.3%) and 32 (10.8%) postoperative complications were reported in the elderly and control group, respectively (p=0.63). Their characteristics are presented in Table-3. Median preoperative-postoperative hemoglobin shift was 1.6 mg% (IQR 1.4), median length of hospitalization (LOH) was 7 days (IQR 3) with no statistical differences between groups. Blood transfusions were carried out for 1 patient (1.8%) in the elderly group, and 4 patients (1.35%) in control group (p=0.58).

**Table 3:** Perioperative complications in elderly and control group.

Complication grade	Elderly group (n = 55)	Control group (n = 296)
Clavien-Dindo 1-2	2 (3.6%)	19 (6.4%)
Clavien-Dindo 3	1 (1.8%) Urinary fistula requiring JJ stent placement - 1	10 (3.4%) Acute urine retention - 2 Bleeding - 4 Pneumothorax - 1 Postoperative hernia - 1 Acute pancreatitis - 1 Bowel obstruction - 1
Clavien-Dindo 4	1 (1.8%) Acute cardiac insufficiency - 1	1 (0.7%) Acute renal failure - 1 Cardiac infarct - 1
Clavien-Dindo 5	0	1 (0.3%) Fatal postoperative bleeding - 1

End-stage renal disease (ESRD) has developed in 10 patients from control group (3.4%) and was not reported in elderly group. When stratified by surgery type, ESRD was more common after RN (6.3%) than after PN (2.8%), but this difference failed to achieve statistical significance. Comparison of perioperative and functional data between elderly patients and younger individuals is summarized in Table-4.

**Table 4:** Perioperative and functional data in elderly and control group.

Variable		Elderly group (n= 55)	Control group (n= 296)	P
**ESRD**				
	(no. / %)	0 / 0%	10 / 3.4%	NS
**Hemoglobin shift**				
	(g/dL, median)	1.6	1.6	NS
**LOH**		7	7	NS
	(days, median)			
**Clavien-Dindo grade ≥3 complications**				
	(no. / %)	2 / 3.6%	13 / 4.4%	NS

**NS** = not significant; **IQR** = interquartile range; **ESRD** = end-stage renal disease; **LOH** = length of hospitalization

## DISCUSSION

With this single-center retrospective analysis we intended to evaluate whether surgery for renal masses is justified in elderly patients. Secondary aims included analysis of perioperative complications and long-term renal function. We found that surgical treatment offers shorter survival in elderly patients with no difference in surgical and functional outcomes.

The process of rapidly aging population results in the consequent increase in the age of hospitalized patients. During the last century life expectancy has increased enormously worldwide. It is estimated that in the next 20 years, the percentage of patients exceeding 85 years may reach 5% (15 million) of people (7). In many of these individuals, renal mass will be found accidentally, requiring decision on further proceeding.

In recent years, we have experienced major changes in surgical treatment of small renal masses. Radical nephrectomy considered previously the gold standard has been gradually replaced with partial nephrectomy. These changes arise from the fact that PN reduces the risk of postoperative renal insufficiency and is associated with longer overall survival with oncologic outcome similar to RN in T1 tumors (8, 9). Along with surgical technique advances, even most-complex tumors like entirely endophytic masses (10) and tumors larger than 4 cm can be treated with nephron-sparing approach (8, 11). Due to aforementioned reasons, partial nephrectomy is currently recommended whenever feasible (12, 13).

In the analyzed cohort, partial nephrectomy constituted 71% of surgeries and was utilized significantly more often in younger patients than in elderly individuals. It can be assumed that surgeons favored RN in older patients due to increased risk of complications and unclear functional benefits of nephron-sparing surgery in this group. Although occurrence of chronic kidney disease, acute renal failure and chronic renal insufficiency has been more likely after RN than PN, first large observational studies failed to prove significant difference in ESRD prevalence depending on surgical approach (14). Recent multi-institutional study on large cohort has however proved that after accounting for individual baseline characteristics, including age and comorbidity, PN protects against need for renal replacement therapy relative to RN (15). In our cohort there was a tendency to lower prevalence of ESRD after PN (6.3% vs 2.8%), but since it failed to achieve statistical significance no clear conclusions can be drawn.

Regardless of surgical approach, oncological outcome of surgery can be influenced by baseline tumor characteristics including stage, grade, histopathologic subtype (16, 17). But for pathologic features, prognostic factors comprise sex, race and individual patients burdens including competing comorbidities and age (16, 18, 19). Although in large cohort studies cancerspecific survival after PN and RN have been shown to exceed 97.5% even in older patients (8), some studies have questioned survival benefit of surgery in patients aged ≥75 years (20, 21). Our data correspond with these findings. We found that overall-survival and cancer-specific survival were significantly better in younger patients. In subgroup analysis elderly patients achieved worse CSS especially in stage ≥T2 disease. Lack of cancer-specific deaths in elderly group and single cancer-specific death in younger group among patients treated with PN can be justified with more restrictive qualification criteria. Patients treated with nephron-sparing surgery had lesions of lower stage. Moreover, it should be noted that only 22 patients aged >75 years underwent nephron-sparing surgery. Due to short-term follow-up in the study, the lack of cancer-specific deaths in this subgroup is not surprising. Simultaneously oncological outcome of RN was substantively diminished in elderly patients when compared to younger individuals (92.4% vs 76.5%).

It has been previously suggested that OS advantage of PN over RN can be modest in older patients. Chung et al. reported that although PN in patients aged ≥65 years was associated with improved renal function when compared to RN, there was no difference in OS (22). Our data seem to support these findings. Younger patients exhibited advantage in overall survival both in general and subgroup analysis. Only in subgroup of PN no difference in survival was detected due to lack of deaths.

Comorbidity and elderly age not only affects oncological and functional results but can also incur postoperative complication (18). Within analyzed group we observed similar complications rate and similar length of hospitalization among elderly and younger patients. However, since elderly patients with multiple comorbidities or chronic kidney disease were *a-priori* disqualified from surgery and partial nephrectomy was utilized less often in older patients, our cohort might differ significantly from previous studies.

The issue of impaired renal function after surgical treatment can be particularly troublesome in elderly individuals. It is questionable whether older patients live long enough to develop end-stage renal disease after surgical treatment for renal cancer. It has been previously observed that although PN in patients ≥65 years was associated with improved renal function when compared to RN, there was no difference in OS and ESRD occurrence (22). In our cohort, among 10 patients who required dialysis, none exceeded 75 years of age. However, it should be noted that within longer follow-up this tendency could change.

Due to disputable outcomes of PN and RN in older patients, the question of overtreatment is unavoidable. One of strategies addressing these issue is to identify most suitable candidates for surgery, achieved with careful selection depending on preoperative characteristics. Based on survival analysis Brassart et al. proposed T-stage, M-stage and Charlson Comorbidity Index as prognostic factors in elderly patients treated with RN (23). Recently, Larcher et al. proposed another SEERderived tool to identify T1 RCC patients who would benefit from the surgery over observation (24). Multivariate model included age, gender, race, Charlson comorbidity index, history of acute kidney injury or chronic kidney disease, tumor size, and year of diagnosis. The benefit of surgery was more visible in younger patients with less comorbidities. Based on 2476 cases selected from SEER database Larcher et al. defined age, comorbidity, acute kidney injury, chronic kidney disease, tumor size and minimally invasive approach as predictors of PN complications (24).

Due to unclear cancer-specific benefits and diminished utility of partial nephrectomy in elderly patients further proceeding in these individuals might differ from standard therapeutic track. Increased cardiovascular mortality reported in previous studies accelerated by renal dysfunction has raised the question of surgical overtreatment in individuals with limited life expectancy (20, 21). Surgery might be deferred (25) or replaced with minimally invasive partial nephrectomy (26) or percutaneous ablation (27, 28). It has been shown that in patients with small renal masses active surveillance is associated with higher quality of life than surgery, having no adverse effects on patients mental health like anxiety or depression (29). In pooled analysis by Smaldone et al. only 18 out of 880 patients (2%) under active surveillance progressed to metastases (30). Sun et al. proved that although nonsurgical management is associated with worse CSS when compared with PN or RN in T1a patients <75 years, in older patients survival benefit is marginal (31). If eventual nephrectomy is indicated, delay in elderly individuals with small renal masses has been suggested to have no impact on cancer-specific survival (25). Short life expectancy constitutes also most significant selection criterium for ablation techniques, which possess excellent functional outcome and low rate of complications (27, 28).

Our study has several limitations that should be mentioned. As with any retrospective study, there is risk for selection bias. All patients were qualified to surgery based on individual patient and surgeon decision, depending on competing comorbidities and individual burdens. For this reason, comorbid patients with short life expectancy were initially disqualified from surgical treatment and not covered by recent analysis. Moreover, elderly patients were more likely to undergo radical nephrectomy than younger patients, which required subgroup analysis to minimize confounding. Another limitation of the study is limited follow-up and significant number of patients lost to follow-up, which might have resulted in suboptimal survival analysis. One can assume that with longer follow-up the differences in overall survival will be more significant with no further difference in cancer-specific survival.

## CONCLUSIONS

Surgical treatment in elderly patients with renal tumor is as safe as in younger individuals and does not increase the risk of ESRD. However, cancer specific survival benefit of PN and RN is significantly diminished in patients aged >75 years with no identified reasons.

## Compliance with Ethical Standards

### Ethical approval

All procedures performed during the study were in accordance with the ethical standards of the institutional and national research committee and with the 1964 Helsinki declaration and its later amendments. For this type of study formal consent is not required.

### Informed consent

Informed consent was obtained from all individual participants included in the study.

### Availability of data and materials

The datasets used and analyzed during the current study are available from the corresponding author on reasonable request.
